# High-Precision Isotopic Analysis of Cu and Fe *via* Multi-Collector Inductively Coupled Plasma-Mass Spectrometry Reveals Lipopolysaccharide-Induced Inflammatory Effects in Blood Plasma and Brain Tissues

**DOI:** 10.3389/fchem.2022.896279

**Published:** 2022-06-15

**Authors:** Kasper Hobin, Marta Costas-Rodríguez, Elien Van Wonterghem, Roosmarijn E. Vandenbroucke, Frank Vanhaecke

**Affiliations:** ^1^ Atomic and Mass Spectrometry - A&MS Research Unit, Department of Chemistry, Ghent University, Ghent, Belgium; ^2^ Barriers in Inflammation Lab, VIB Center for Inflammation Research, Ghent, Belgium; ^3^ Department of Biomedical Molecular Biology, Ghent University, Ghent, Belgium

**Keywords:** Cu, Fe, blood plasma, brain, isotopic analysis, MC-ICP-MS, inflammation, LPS-infected mice

## Abstract

The concentration and the isotopic composition of the redox-active essential elements Cu and Fe were investigated in blood plasma and specific brain regions (hippocampus, cortex, brain stem and cerebellum) of mice to assess potential alterations associated with sepsis-associated encephalopathy induced by lipopolysaccharide (LPS) administration. Samples were collected from young (16–22 weeks) and aged (44–65 weeks) mice after intraperitoneal injection of the LPS, an endotoxin inducing neuroinflammation, and from age- and sex-matched controls, injected with phosphate-buffered saline solution. Sector-field single-collector inductively coupled plasma-mass spectrometry was relied upon for elemental analysis and multi-collector inductively coupled plasma-mass spectrometry for isotopic analysis. Significant variations were observed for the Cu concentration and for the Cu and Fe isotope ratios in the blood plasma. Concentrations and isotope ratios of Cu and Fe also varied across the brain tissues. An age- and an inflammatory-related effect was found affecting the isotopic compositions of blood plasma Cu and cerebellum Fe, whereas a regional Cu isotopic redistribution was found within the brain tissues. These findings demonstrate that isotopic analysis of essential mineral elements picks up metabolic changes not revealed by element quantification, making the two approaches complementary.

## Introduction

Inflammation is the response of the immune system triggered by invading pathogens, damaged cells and/or toxic compounds. Studies on inflammation mechanisms and its biological role are crucial for understanding physiological immune responses and pathologies. Sepsis-associated encephalopathy (SAE), *i.e.* brain dysfunctioning as a result of peripheral infection, is often observed in sepsis and is associated with increased mortality in sepsis patients. Dysregulation of inflammatory responses in the central nervous system (CNS), *i.e.* neuroinflammation, is believed to play a major role during the pathogenesis of SAE (Zampieri et al., 2011; Ren et al., 2020). Neuroinflammation has also been accepted as an important factor during the pathogenesis in neurological disorders (NDs) such as Alzheimer’s disease (AD), Parkinson’s disease (PD), and amyotrophic lateral sclerosis (ALS) (Schain and Kreisl, 2017).

The CNS is separated from the peripheral system by two highly selective semipermeable barriers, the blood brain barrier (BBB) and the blood cerebrospinal fluid barrier (BCSFB). The BBB and BCSFB consist of endothelial cells and choroid plexus epithelial cells, respectively, forming tight junctions restricting blood-borne substances from entering the CNS (De Bock et al., 2014). The permeability of the BBB and BCSFB can change in response to numerous factors, such as trauma, medication and stress. Sepsis-induced dysregulated host response causes endothelial activation, which results in increased endothelial permeability (Sharshar et al., 2010). Increased permeability of the BBB allows proinflammatory cytokines from the periphery, released in response to the infection, to cross the BBB and activate microglia leading to neuroinflammation and loss of neuronal function (Nwafor et al., 2019). Additionally, the choroid plexus epithelium, forming the BCSFB, is prone to leakiness upon peripheral infection due to matrix metalloproteinase activity (Vandenbroucke et al., 2012). Impaired brain barriers have also been observed in ageing, making elderly people more susceptible to neuroinflammation (Stephenson et al., 2018).

The administration of LPS *in vivo* is a well-established procedure for the activation of the immune system in murine models (Nava Catorce and Gevorkian, 2016). LPSs are large molecules found at the outer membrane of Gram-negative organisms and are composed of three major components: an O-antigen with multiple repeated units of monosaccharides, an oligosaccharide core and lipid A, which is composed of a diglucosamine backbone with six fatty acid chains attached to it (Domínguez-Medina et al., 2020). The lipid A domain is responsible for the toxicity of a LPS when released into circulation, causing fever, diarrhea and a possible fatal septic shock (Raetz and Whitfield, 2002; Dutta et al., 2008). After intraperitoneal injection, LPS interacts with the LPS-binding protein and CD-14, a co-receptor anchored on the outer membrane of peripheral immune cells (macrophages and monocytes), after which interaction with transmembrane Toll-like receptor-4 activates various intracellular signaling pathways (Raetz and Whitfield, 2002; Dutta et al., 2008). Activated peripheral immune cells produce and release proinflammatory cytokines (e.g., Interleukin (IL)-1, IL-6, tumor necrosis factor (TNF)-α) and reactive oxygen species (ROS), leading to inflammation (Deng et al., 2017; Stephenson et al., 2018).

Oxidative stress is the result of an imbalance between ROS and anti-oxidants in a biological system. These systems may become overwhelmed during neuroinflammation as levels of pro-oxidants rise (Greenough et al., 2013). ROS are produced by direct interactions between ions of unbound redox-active metals (e.g., Cu and Fe) and oxygen species (H_2_O_2_ and O_2_^•^) via Fenton and Haber-Weiss chemistry (Barnham et al., 2004; Valko et al., 2005; Greenough et al., 2013). In the body, Cu and Fe are transported and stored bound to proteins, such as albumin and ceruloplasmin (Cp) (transport) and metallothionein (storage) for Cu and transferrin (transport) and ferritin (storage) for Fe. Dyshomeostasis of these metals during neuroinflammation upregulates ROS production and oxidative stress. Several hypotheses regarding the factors governing the dysregulation of trace elements in NDs have been put forward, such as BBB dysfunctioning, decreased metal clearance and/or dysregulation of metal transport proteins [e.g., ferroportin] (Nemeth et al., 2004; Sharshar et al., 2010; Vandenbroucke et al., 2012).

Variations in the isotopic composition of essential metals (Cu and Zn) associated to NDs have been reported in different body organs and fluids (Mahan et al., 2018; Moynier et al., 2020; Vanhaecke and Costas-Rodríguez, 2021). Differences in the isotopic composition of an essential mineral element between body compartments occurs as a result of isotope fractionation accompanying biochemical processes. The occurrence of isotope fractionation may be linked to (very slight) differences in reaction rate between the isotopes and differences in the bonding environment and redox state, e.g., Cu(I)/Cu(II) and Fe(II)/Fe(III), between compartments (Balter et al., 2013). The presence of different redox states for Cu and Fe enlarges their isotopic variation in biological systems. In a previous work, the isotopic composition of the non-redox active metal Mg was investigated together with the concentration of some essential elements in the LPS murine model. A heavy Mg isotopic composition and elevated Mg and P levels were observed in blood plasma of young and aged LPS-injected mice compared to the matched controls. The Mg isotopic composition was also altered in the liver, urine and brain stem of the aged mice (Grigoryan et al., 2021).

In the context of AD, the blood plasma and brain Fe isotopic compositions of tau-transgenic L66 mice (tau-based animal model) were demonstrated to be lighter than those of the matched controls (Solovyev et al., 2021). However, no significant changes were observed in the blood plasma/serum and brain Cu isotopic compositions, neither in tau-transgenic L66 mice, nor in two amyloid-beta (Aβ)-based animal models [*i.e.* AAPswe/PSEN1dE9 mice and APP/PSEN1 (5xFAD) transgenic mice] (Moynier et al., 2019; Solovyev et al., 2021), compared to the corresponding matched controls. In an ALS murine model (transgenic mice expressing the G93A mutant form of human SOD1), Cu isotope ratio data in various body compartments (spinal cord, brain, muscle and blood) did not show a significant difference compared to these in the matched controls (Enge et al., 2017). In humans, the Cu isotopic composition of CSF from patients suffering from ALS was significantly heavier than that from the age-matched controls and patients suffering from AD (Sauzéat et al., 2018). The study of metal homeostatic alterations in (neuro)inflammation *via* high-precision isotopic analysis of redox-active elements, such as Cu and Fe, thus can provide relevant information concerning the role of these elements in ageing, SAE and NDs pathogenesis.

In this work, LPS (in phosphate buffered saline solution–PBS) was injected intraperitoneally in young (16–22 weeks) and aged (44–65 weeks) mice to investigate potential changes in redox-active metal (Cu and Fe) concentrations and isotope ratios related to the effect of sepsis and SAE. Observed changes in metal concentrations and isotope ratios could potentially contribute to a better understanding of the role of these redox-active metals during peripheral and neuroinflammatory responses. Age- and sex-matched controls were injected with PBS. Blood plasma and brains were collected after perfusion to remove blood contamination. Different brain parts were isolated *post mortem*–cortex, brain stem, hippocampus and cerebellum–for further evaluation of possible regional differences. Elemental and isotopic analysis, performed *via* sector-field single-collector inductively coupled plasma-mass spectrometry (SF-ICP-MS) and multi-collector inductively coupled plasma-mass spectrometry (MC-ICP-MS), respectively, were relied upon to reveal potential variations in metal concentrations and isotope ratios resulting from the dysregulation of metal homeostasis.

## Materials and Methods

### Reagents

Trace metal analysis grade 14 M HNO_3_ and 12 M HCl acquired from PrimarPlus (Fisher Chemicals, UK) were further purified via sub-boiling distillation in a Savillex® DST-4000 acid purification system (Savillex Corporation, United States). TraceSELECT® 9.8 M H_2_O_2_ was acquired from Sigma-Aldrich (Belgium). Ultrapure water with a resistivity ≥18.2 MΩ cm was obtained from a Milli-Q Element water purification system (Millipore, France) and will further be referred to as MQ-water. The isotopic reference materials used for the isotope ratio measurements were NIST SRM 976 for Cu (National Institute of Standards and Technology–NIST, United States) and IRMM-014 for Fe (Institute for Reference Materials and Measurements–IRMM, Belgium). Adequate dilutions and mixtures of 1 g.L^−1^ single-element standard stock solutions of Cu, Fe, Ni and Ga (Inorganic Ventures, the Netherlands) were used for quantification, mass bias correction (Ni and Ga as admixed internal standards), and for checking the quality of the isotope ratio measurements (Cu and Fe in-house standards). Sample preparation was performed in a class-10 clean lab (PicoTrace, Germany).

### Samples

Blood and brain samples were obtained from mice housed in the SPF facility at the VIB Center for Inflammation Research. Mice were kept in individually ventilated cages under a 12-h dark/12-h light cycle in a specific pathogen-free animal facility and received food and water *ad libitum*. All animal studies were conducted in compliance with governmental and EU guidelines for the care and use of laboratory animals and were approved by the ethical committee of the Faculty of Sciences, Ghent University, Belgium. Wild type (C57Bl/6 background) male mice were injected intraperitoneally with LPS (10 mg/kg body weight) to induce systemic inflammation. Because differences in the Fe isotope ratios could occur between male and female mice, one murine model of genetically identical mice with the same sex was used to focus on the effects of sepsis and inflammatory effects on Fe and Cu isotope ratios (Jaouen and Balter, 2013; Van Heghe et al., 2013). LPS-injection was performed with a group of young (16–22 weeks old, N = 10) and a group of aged (44–65 weeks old, N = 10) mice. Twenty-four hours after administration of the LPS, the animals were sacrificed. Body temperature was continuously monitored and the animals were euthanized earlier when exhibiting severe hypothermia (*i.e.* a body temperature below 28°C). In addition, a group of young (N = 15) and a group of aged (N = 10) male mice injected with a saline (PBS) solution and sacrificed 24 h after injection were used as controls. Blood plasma and brains were collected after perfusion from each individual. From the brain, four different parts were collected *post mortem* for further analysis: cortex, brain stem, hippocampus and cerebellum. Immediately after collection, the samples were stored at −20°C until sample preparation commenced.

### Sample Preparation

Blood plasma and brain tissues were accurately weighed in pre-cleaned Savillex® beakers and 2 ml of 14 M HNO_3_ and 0.5 ml of 9.8 M H_2_O_2_ were added. The beakers were kept closed on a hotplate for 16 h at 110 °C to ensure complete mineralization of the samples. The digests thus obtained were evaporated to dryness and the residues were re-dissolved in 5 ml of 8 M HCl + 0.001% H_2_O_2_.

Chromatographic isolation of the target elements was carried out using analytical grade AG® MP-1 strong anion exchange resin (100–200 µm dry mesh size, chloride anionic form, Bio-Rad, Belgium) packed in polypropylene chromatographic columns (Eichrom, France). The resin was first pre-cleaned with 10 ml of 7 M HNO_3_, 10 ml of MQ-water, 10 ml of 0.7 M HNO_3_ and 10 ml of MQ-water and conditioned with 5 ml of 8 M HCl + 0.001% H_2_O_2_. The sample was loaded onto the column and the matrix was eluted using 3 ml of 8 M HCl + 0.001% H_2_O_2_. Afterwards, Cu and Fe were eluted successively using 9 ml of 5 M HCl + 0.001% H_2_O_2_ and 7 ml of 0.7 M HCl and collected in Teflon Savillex® beakers. The purified fractions of Cu and Fe thus obtained were evaporated to dryness at 90°C. The Cu fractions were taken up and subjected to a second chromatographic isolation following the same steps for removal of remaining Na (Costas-Rodríguez et al., 2015). Two steps of evaporation were carried out to remove residual chlorides from the Cu and Fe fractions. The final residues were re-dissolved in 500 µL of 0.28 M HNO_3_ for further elemental and isotopic analysis. Two procedural blanks treated in the same way as the samples were included in each batch of samples. The blank contributions were determined to be ≤ 1 and ≤ 4% for Cu and Fe, respectively.

### Instrumentation and Measurements

Elemental determinations were performed *via* SF-ICP-MS. The instrument used is a Thermo Scientific Element XR (Germany), equipped with a 200 μL min^−1^ quartz concentric nebulizer mounted onto a cyclonic spray chamber for sample introduction into the plasma. Quantification was performed *via* external calibration, with Ga as an internal standard (10 µg.L^−1^) to correct for potential matrix effects and/or instrument instability. Cu and Fe isotope ratio measurements were carried out using a Thermo Scientific Neptune *Plus* MC-ICP-MS instrument (Germany), equipped with a high-transmission jet interface. Sample introduction was accomplished using a 100 μL min^−1^ PFA concentric nebulizer mounted onto a dual spray chamber, consisting of a cyclonic and a Scott-type sub-unit. All isotope ratio measurements were performed 1) at medium (pseudo) mass resolution, 2) in static collection mode, involving Faraday collectors connected to 10^11^ Ω amplifiers, and 3) on the interference-free plateau to the left of the peak center. The instrument settings and data acquisition parameters are summarized in Table 1. Correction for mass discrimination was performed *via* the combination of 1) internal correction by means of a linear regression line in ln-ln space and Russell’s equation and 2) external correction in a sample-standard bracketing approach (Woodhead, 2002; Baxter et al., 2006). For internal correction, Ni and Ga were relied on as internal standard for Fe and Cu, respectively. The isotope ratios obtained are reported in delta notation, δ^56^Fe, and δ^65^Cu (‰), following Equations (1), (2),

δ5xFe= (( 5xFe 54Fe)sample( 5xFe 54Fe)standard−1)x 1000‰ (1)

x is six or seven

δ65Cu= (( 65Cu 63Cu)sample( 65Cu 63Cu)standard−1)x 1000‰ (2)

using the isotopic reference materials IRMM-014 and NIST SRM 976 as external standard for Fe and Cu, respectively.

**TABLE 1:** Instrument settings and data acquisition parameters for SF-ICP-MS and for MC-ICP-MS.

Instrument Settings	Element XR	Neptune *Plus*
Sample uptake rate (µL min^−1^)	200	100
Plasma gas flow rate (L min^−1^)	15	15
Auxiliary gas flow rate (L min^−1^)	0.80	0.7–0.8
Nebulizer gas flow rate (L min^−1^)	1.050	1.0–1.1
Rf Power (W)	1,250	1,200
Guard electrode	Connected	Connected
Sampling cone	Ni, 1.1 mm orifice diameter	Ni, Jet cone, 1.1 mm orifice diameter
Skimmer	Ni, 0.8 mm orifice diameter	Ni, X-type, 0.8 mm orifice diameter
Resolution mode	Medium	Pseudo-medium
**Data acquisition parameters**	**Element XR**	—
Acquisition mode	E-scan	—
Number of runs and passes	5 × 5	—
Dwell time per point (ms)	10	—
Points per peak	20	—
Nuclides monitored	^63^Cu,^65^Cu,^54^Fe,^56^Fe,^69^Ga	—
**Data acquisition parameters**	—	**Neptune *Plus***
Scan type	—	Static, multicollection
Number of blocks and cycles	—	9 × 5
Integration time (s)	—	4.194
Cup configuration—Cu (Ga as IS)	—	L4:^63^Cu, L2:^65^Cu, C:^67^Zn H2:^69^Ga, H4:^71^Ga
Cup configuration—Fe (Ni as IS)	—	L4:^54^Fe, L2:^56^Fe, L1:^57^Fe H1:^60^Ni, H3:^62^Ni

### Statistical Analysis

Statistical tests were performed using the IBM^®^ SPSS Statistics 28 software for Windows. The Shapiro-Wilk test was used to evaluate the normality of the data. The independent samples *t*-test was applied for parametric data, whereas for non-parametric data, the Kruskal–Wallis and Mann-Whitney U tests were relied on to evaluate whether the means or medians of two independent groups are significantly different. A *p* ≤ 0.05 level of significance was chosen as a threshold value for statistical evaluation.

## Results

### Blood Plasma

**FIGURE 1:**
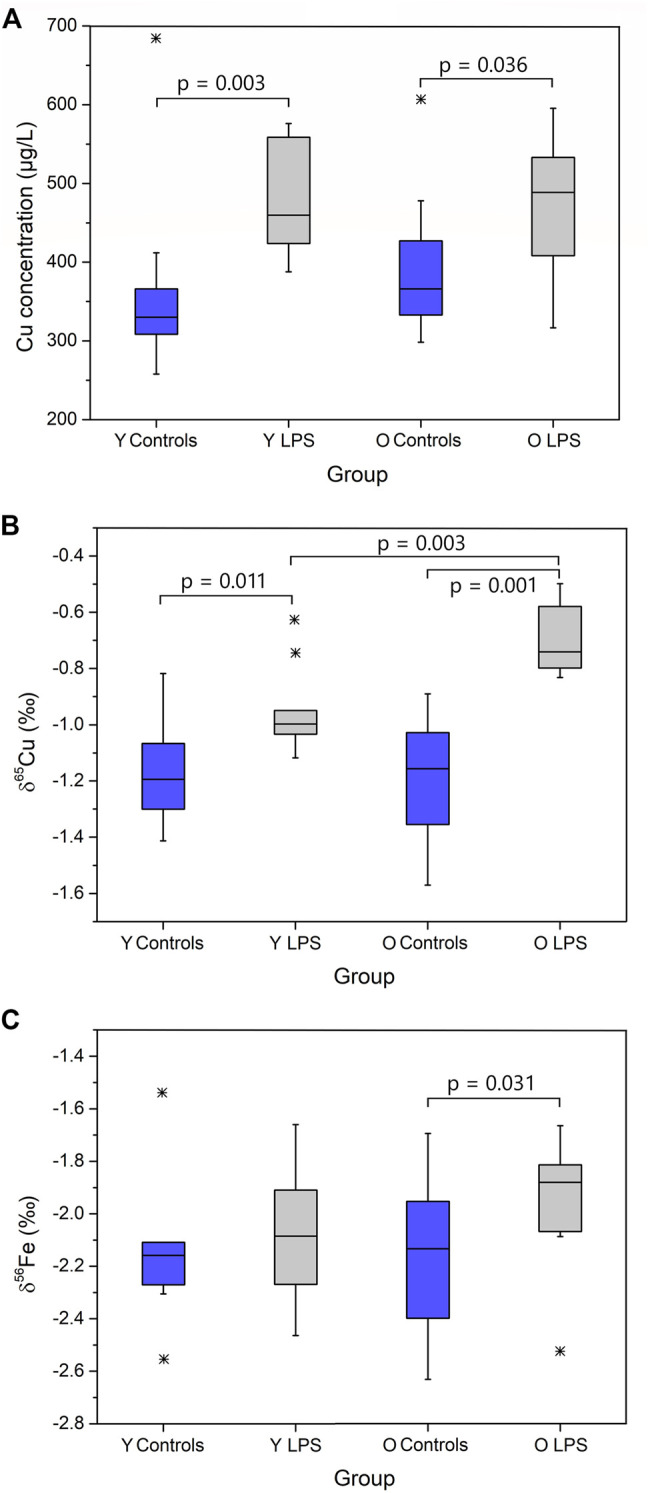
Plasma Cu concentration **(A)**, δ^65^Cu value **(B)** and δ^56^Fe value **(C)** for young and aged LPS-injected mice and matched controls. Y LPS, O LPS, Y Controls and O Controls correspond to the young and aged infected mice and young and aged controls, respectively. Outliers were indicated by a star. Only *p*-values that indicate a significant difference between populations (*p* ≤ 0.05) are shown.

The concentrations and isotope ratios of Cu and Fe in blood plasma of the LPS-injected mice and controls are summarized in Table 2. The data are expressed as median and interquartile range (IQR). N is the number of individuals in each group. Plasma Fe concentrations were not significantly different between the groups of mice. The LPS injection resulted in elevated plasma Cu concentrations (Mann-Whitney U test; *p* < 0.05) for both young and aged individuals (Mann-Whitney U test; *p* < 0.05) compared to the matched controls. Cu blood plasma concentrations are shown in Figure 1A.

**TABLE 2:** Blood plasma concentrations and isotope ratios of Cu and Fe in young and aged LPS-injected mice and matched controls. Y LPS, O LPS, Y Controls and O Controls correspond to the young and aged infected mice and young and aged controls, respectively. N is the number of individuals.

Sample Type	Cu Concentration (10^2^ μg.L^−1^)			δ^65^Cu _NIST SRM 976_ (‰)		
	Median	IQR	N	Median	IQR	N
Y Controls	3.3	0.5	10	−1.19	0.21	10
Y LPS	4.6	1.2	10	−1.00	0.08	10
O Controls	3.7	0.8	15	−1.16	0.30	14
O LPS	4.9	1.2	10	−0.74	0.20	10
	**Fe concentration (10** ^**2**^ ** μg.L** ^**−1**^ **)**			**δ** ^**56**^ **Fe**_**IRMM-014**_ **(‰)**		
**—**	**Median**	**IQR**	**N**	**Median**	**IQR**	**N**
Y Controls	7.7	7.9	10	−2.16	0.14	8
Y LPS	7.4	4.9	10	−2.08	0.36	9
O Controls	6.5	4.5	15	−2.13	0.40	15
O LPS	10.3	9.4	10	−1.88	0.23	10

The plasma Cu isotopic composition of the LPS-injected mice was significantly heavier than that of the controls for both young and aged mice (Mann Whitney U test, *p* < 0.05) (Figure 1B). Moreover, the enrichment in the heavy ^65^Cu isotope was more pronounced (+0.4‰) in the aged mice than in the young mice (+0.2‰). In Figure 1C, the plasma Fe isotopic composition in the young and aged LPS-injected mice and matched controls are shown. As can be seen, the Fe isotopic composition was significantly heavier in the aged mice only (Mann-Whitney U test, *p* < 0.05).

### Brain Tissues

The concentrations and the isotope ratios of Cu and Fe in different brain regions (hippocampus, cortex, cerebellum and brain stem) for the four groups of mice, *i.e.* young and aged LPS-injected mice and matched controls, are shown in Table 3. Data are expressed as median and interquartile range (IQR). N is the number of individuals in each group. Within the brain, the highest Cu concentration was found in the cerebellum, whereas the other brain regions showed similar values. Moreover, the Cu concentration was slightly higher in the cerebellum of the aged mice compared to that in the young mice. Fe concentrations in the brain tissues showed a large biological variability and no clear trends were found.

**TABLE 3:** Cu and Fe concentrations and δ^65^Cu and δ^56^Fe values obtained for the hippocampus, cortex, brain stem and cerebellum for the young and aged LPS-injected mice and matched controls. Y LPS, O LPS, Y Controls and O Controls correspond to the young and aged infected mice and young and aged controls, respectively. N is the number of individuals.

		Cu (µg g^−1^)			Fe (µg g^−1^)			δ^65^Cu (‰)			δ^56^Fe (‰)		
		Median	IQR	N	Median	IQR	N	Median	IQR	N	Median	IQR	N
**Y Controls**	Hippocampus	3.71	0.72	5	5.83	2.37	5	0.34	0.26	5	−2.27		2
	Cortex	2.96	0.28	5	6.59	4.90	5	0.17	0.14	5	−2.19	0.43	4
	Brain stem	3.44	0.91	5	5.23	2.54	5	0.42	0.20	5	−1.93	0.35	4
	Cerebellum	5.18	0.07	5	9.54	8.02	5	0.40	0.41	5	−2.10	0.06	3
**Y LPS**	Hippocampus	3.44	1.19	5	4.45	4.77	5	0.32	0.17	5	−2.16	0.23	4
	Cortex	3.16	0.97	5	4.91	5.08	5	0.01	0.18	5	−2.06	0.14	5
	Brain stem	3.35	0.89	5	4.33	4.87	5	0.38	0.10	5	−2.14	0.11	5
	Cerebellum	4.95	0.34	5	4.16	9.66	5	0.25	0.18	5	−2.14	0.14	5
**O Controls**	Hippocampus	3.07	0.31	5	8.79	5.15	5	0.27	0.06	3	−2.29		2
	Cortex	2.79	0.99	5	5.22	4.63	5	0.11	0.16	5	−2.34	0.10	4
	Brain stem	3.25	0.60	5	4.08	0.20	5	0.33	0.24	5	−2.19	0.14	4
	Cerebellum	6.13	0.29	5	6.58	6.62	5	0.25	0.29	5	−2.50	0.05	5
**O LPS**	Hippocampus	3.76	1.02	5	5.70	15.54	5	0.32	0.08	5	−2.79	0.45	5
	Cortex	3.35	0.32	5	4.83	12.03	5	0.14	0.19	5	−2.20	0.47	5
	Brain stem	3.48	0.54	5	3.79	0.07	5	0.33	0.19	5	−2.28	0.18	5
	Cerebellum	6.09	2.09	5	6.03	1.29	5	0.24	0.27	5	−2.35	0.08	5

The Cu isotopic composition was heterogeneous within the brain (Table 3). The cortex showed the lightest (lowest δ^65^Cu value) and the brain stem the heaviest (highest δ^65^Cu value) Cu isotopic composition, respectively, for both LPS-injected mice and controls. In general, the δ^65^Cu values tend to be systematically lighter in the LPS-injected mice, although when comparing to the control values, the level of significance was not reached.

**FIGURE 2:**
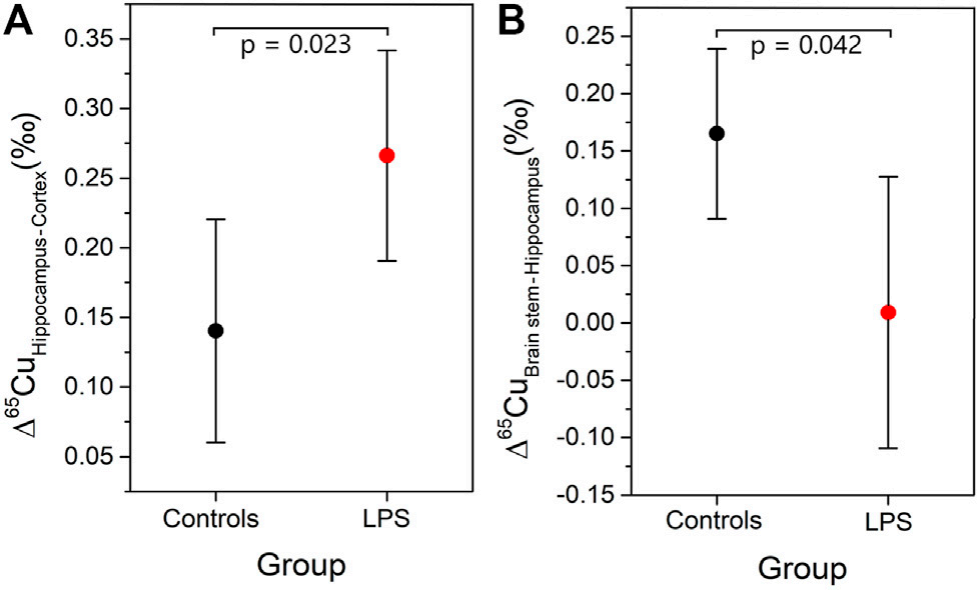
Mean and 95% confidence interval for Δ^65^Cu values between different regions in the brain **(A)** Δ^65^Cu_Hippocampus-Cortex_ and **(B)** Δ^65^Cu_Brain stem-Hippocampus_.

As an age effect was not observed in the Cu isotopic composition in any of the brain regions, the young and aged individuals were grouped for further evaluation. The difference in the δ^65^Cu values between brain regions was determined and expressed as capital delta [Δ^65^Cu, equations (3) and (4)]. The Δ^65^Cu values showed a parametric distribution (Shapiro-Wilk test) and thus, the independent samples *t*-test was applied to evaluate the significance of the difference between the means (*p* < 0.05). Figure 2 shows the Δ^65^Cu values obtained for the combinations hippocampus-cortex and brain stem-hippocampus. In the controls, the difference between the averages for the hippocampus and the cortex was 0.15‰. However, this value increased significantly to a difference of 0.26‰ in the LPS-injected mice. While the difference between the hippocampus and cortex increased upon LPS injection (independent samples *t*-test, *p* < 0.05), an opposite effect was observed between the brain stem and hippocampus (independent samples *t*-test, *p* < 0.05). No significant effects were observed for the Δ^65^Cu values between other brain regions.

$${\text{Δ}}^{65}Cu_{Hippocampus-Cortex}\;\left(‰\right)=\;\delta^{65}Cu_{Hippocampus}\;\left(‰\right)-\;\delta^{65}Cu_{Cortex}\;\left(‰\right)$$ (3)

$${\text{Δ}}^{65}Cu_{Brain\;stem-Hippocampus}\;\left(‰\right)=\;\delta^{65}Cu_{Brain\;stem}\;\left(‰\right)-\;\delta^{65}Cu_{Hippocampus}\;\left(‰\right)$$ (4)

**FIGURE 3:**
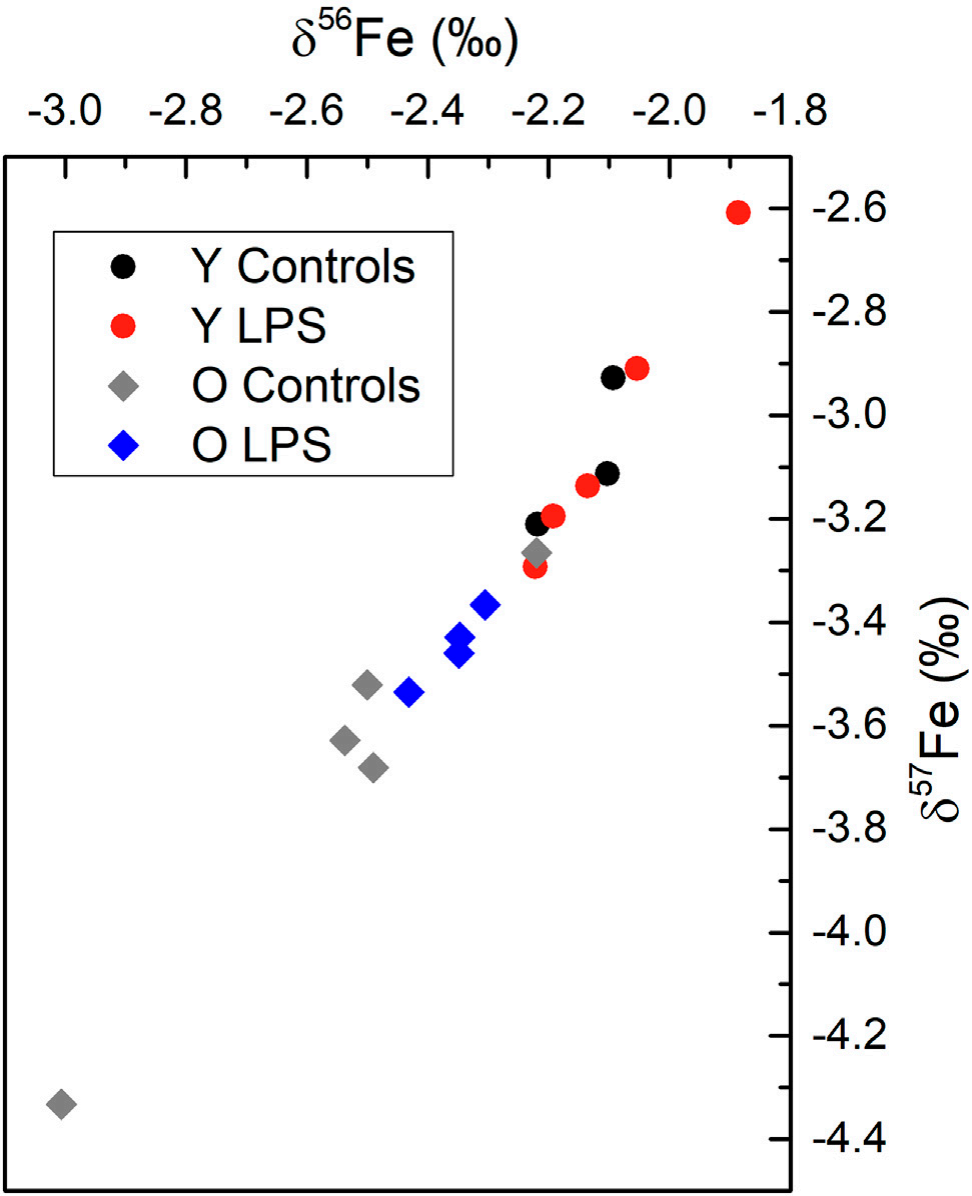
Three-isotope plot for Fe for the cerebellum combining data for each group of mice. Y LPS, O LPS, Y Controls and O Controls correspond to the young and aged infected mice and young and aged controls, respectively. The measurement precision expressed as two SD was 0.03‰ for δ^56^Fe and 0.07‰ for δ^57^Fe.

The Fe isotopic composition showed a wide variability among different parts of the brain and among individuals. The variability observed in δ^56^Fe values for the different brain tissues of a single individual ranged between 0.03 ‰ and 1.43‰. The variability observed within the same brain tissue for different individuals ranged between 0.13 ‰ and 1.36‰. Between the different brain sections, no systematic patterns were observed. Additionally, for the hippocampus, brain stem and cortex no systematic differences were observed between the young and aged and LPS-injected and healthy mice. In spite of this, a significant effect was observed for the Fe isotopic composition of the cerebellum. Figure 3 shows the three-isotope plot of the Fe isotopic composition of the cerebellum for the different groups. On the one hand, the aged mice showed a significantly lighter cerebellum Fe isotopic composition compared to that of the young individuals (Mann-Whitney U test, *p* < 0.05). On the other hand, the aged LPS-injected mice tend towards a heavier cerebellum Fe isotopic composition compared to the matched controls, although it remains lighter than that of the young mice (both, with and without LPS injection).

## Discussion

Elevated Cu concentrations observed in blood plasma for both young and aged LPS-injected mice, compared to controls, are in good agreement with previous findings in serum/plasma for humans and animal models under inflammatory conditions, oxidative stress and ageing (Milanino et al., 1993; Guo et al., 2011). It has been demonstrated that the inflammatory stimuli elevate Cu plasma concentrations by affecting the synthesis of carrier proteins for Cu in serum, e.g., ceruloplasmin (Cp), on the hepatic level (Milanino et al., 1993; Malavolta et al., 2015). Therefore, the serum Cu concentration is elevated in response to the LPS-injection *via* a hepatically orchestrated mechanism. Some cytokines relevant in the acute phase response, e.g., IL-6, IL-1β, TNF-α and interferon-γ (IFN-γ), have been reported to be elevated in the plasma of LPS-injected mice (Wan et al., 1995; Remick et al., 2000). These inflammatory biomarkers are known to upregulate the synthesis of Cp, the major transport protein of Cu in blood plasma (Sidhu et al., 2011). In humans, elevated levels of Cu in peripheral plasma were also found associated to NDs (Arnal et al., 2010).

Also the blood plasma Cu isotope ratio was significantly altered in the LPS-injected mice (both young and aged mice), showing an enrichment in the heavy ^65^Cu isotope compared to the controls. Moreover for the aged mice, this enrichment (+0.4‰) was more pronounced than for the young mice (+0.2‰). This pattern seems to be consistent with the higher vulnerability of aged mice to LPS infection (Starr et al., 2010). This effect, although still poorly understood, seems to be associated to the breakdown of anatomic barriers, underlying illnesses and/or the declining of the immune system (Tateda et al., 1996; Vandenbroucke et al., 2012; Gorlé et al., 2016; Nwafor et al., 2019). Alterations in protein expression are believed to affect the blood plasma Cu isotopic composition, as it has been evidenced in prion protein transgenic mice (Büchl et al., 2008; Miller et al., 2016). Thus, the heavy blood plasma Cu isotopic composition of the LPS-injected mice could be associated to alterations in Cu-binding proteins. As the majority of the serum Cu (60%–95%) is bound to Cp, alterations in the expression of the latter could plausibly induce noticeable changes in the plasma Cu isotopic composition. Also, the Cu isotopic redistribution in the body, accumulation of Cu in different organs (which preferentially take up the lighter ^63^Cu isotope) and changes in absorption and excretion of dietary Cu could potentially explain the heavy plasma Cu isotopic composition of the LPS-injected mice compared to controls. LPS was found to stimulate the Cu uptake by increasing the expression of the copper importer CTR1. Little is known about the mechanisms altering Cu homeostasis within specific organs in response to infection, but it is accepted that Cu is mobilized towards sites of infection for bacterial killing by macrophages (Hodgkinson and Petris, 2012; Subirana et al., 2022).

The blood plasma Fe isotopic composition was only significantly heavier in aged mice after LPS-injection compared to controls. Iron homeostasis is regulated by systemic control mechanisms of Fe conservation and intestinal absorption and is tightly connected to inflammation and/or infection response. Reduced Fe absorption and increased intracellular sequestration of the metal are associated with inflammatory conditions (Nemeth et al., 2003). Previous works evidenced that the serum/whole blood Fe isotopic composition reflects an individual´s Fe status (Hotz and Walczyk, 2013; Van Heghe et al., 2013). Individuals with low Fe status and upregulated intestinal absorption show a heavy blood/serum Fe isotopic composition and *vice versa* (Krayenbuehl et al., 2005; Anoshkina et al., 2017). Cytokines, hepcidin and other acute phase proteins are responsible for limiting circulating Fe or sequestering it from microorganisms. Systemic infections are associated to a host metabolic response, restricting pathogen access to Fe (Soares and Weiss, 2015). The large majority of the Fe available for the pathogens originates from hemoglobin (Gozzelino and Soares, 2014). Upon hemolysis, extracellular hemoglobin is oxidized and released to the plasma (Pamplona et al., 2007; Larsen et al., 2010). The fact that a relevant effect on the Fe isotopic composition was observed in the aged mice only, could be associated to the vulnerability of the immune system in the aged mice to the LPS, the resistance to the infection and/or the inflammatory status (Starr et al., 2010).

In order to evaluate possible regional differences in the brain upon inflammation induced by the LPS injection, the Cu and Fe concentrations and isotope ratios were explored in the hippocampus, brain stem, cerebellum and cortex. Differences in the regional metal distribution observed between healthy and LPS-injected individuals, *i.e.* elevated levels of Cu in the cerebellum, are consistent with the results obtained earlier via quantitative elemental analysis and/or mapping techniques of human and animal brains (Popescu et al., 2009). Rajan *et al.* reported a Cu concentration of 7.4 ± 2.5 μg/g in the cerebellum of healthy individuals, which is in good agreement with the data obtained in this study (Rajan et al., 1997). Copper homeostasis in the CNS takes place via the absorption, accumulation and excretion processes, which are primarily located at the BBB (Zheng and Monnot, 2012). Copper transport proteins include divalent metal transporter 1, copper transporter-1 and copper-transporting P-type ATPases, ATP7A and ATP7B (Harris, 2001). Copper homeostasis was reported to be affected under inflammatory conditions due to secretion of IFN-γ by natural killer cells, which stimulates ATP7A expression with Cu sequestration in microglia as a result (Solerte et al., 2000). Moreover, Wei *et al.* reported a higher level of IFN-γ in the cerebellum of aged mice compared to young mice (Wei et al., 2000). Therefore elevated Cu concentrations in the cerebellum of aged mice could be related to an inflammatory and age effect, resulting in an increased sequestration of Cu in glial cells.

The Cu isotopic composition of the different brain tissues was about 1–1.2‰ heavier than that of the blood plasma. Moynier et al. (2019) reported a difference of 1‰ between brains and serum samples of a mouse model of AD and controls which is consistent with our results. Miller et al. (2016) observed the lightest Cu isotopic composition in the cortex and the heaviest in the brain stem of wild type and prion protein transgenic mice. This trend is similar to the results obtained for LPS-injected mice and controls. Overall, a lighter Cu isotopic composition was found in brain tissue of the LPS-injected mice compared to that in the controls. In the brain, Cu is mainly found either bound as Cu(I) to glutathione and metallothionein or present as Cu(II) in the synapses (Scheiber et al., 2014). Based on calculations of fractionation factors for Cu(I) and Cu(II), the reduced form Cu(I) is expected to be isotopically lighter than the Cu(II) form (Moynier et al., 2017). The lighter Cu isotopic composition of the brain tissues in the LPS-injected mice could be related to degeneration of neuronal cells and the loss of synaptic activity.

The Cu isotopic differences observed between the hippocampus/cortex and the brain stem/hippocampus indicate a Cu isotopic redistribution between the aforementioned brain regions (Figure 2). The redistribution in the brain observed for the LPS-injected mice can be related to the disruption of the BBB/BCSFB and/or to brain leakiness (Sharshar et al., 2010; Vandenbroucke et al., 2012). Further research of the BBB and BCSFB integrity in brain tissues is required to confirm these hypotheses.

In the cerebellum, no changes in Fe concentrations were observed, but Fe isotope ratios showed two trends; 1) aged mice showed a significantly lighter cerebellum Fe isotopic composition compared to that of the young individuals, and 2) LPS-injection in aged mice was observed to affect the Fe isotopic composition towards a heavier isotopic composition than that of the matched controls although it remains lighter than that of the young mice. The level of significance was not reached within the limited data set. These results suggest that ageing causes Fe isotopic variations in the cerebellum, whereas inflammation seems to affect the Fe isotopic composition of the aged mice only.

## Conclusion

MC-ICP-MS and SF-ICP-MS were relied upon to investigate biological changes related to sepsis and SAE reflected in the concentrations and isotope ratios of redox-active metals.

LPS injection resulted in increased Cu blood plasma levels for both young and aged individuals compared to controls. This effect is presumably related to the inflammatory response. The blood plasma Cu isotopic composition of the LPS-injected mice was significantly altered for both young and aged mice, while the Fe isotopic composition was only significantly altered in the aged LPS-injected mice, probably due to a higher vulnerability of the aged mice to the LPS. Both effects are presumably related to changes in protein expression, isotopic redistribution and/or changes in absorption and excretion as a result of the LPS administration.

The highest Cu concentration levels were observed in the cerebellum, an observation that was even more pronounced for the aged mice. Both redox-active elements (Cu and Fe) displayed a heterogeneous isotopic distribution within the brain. The cortex and the brain stem showed the lightest and the heaviest δ^65^Cu values, respectively. A Cu isotopic redistribution was found between hippocampus and cortex, and between brain stem and hippocampus in the LPS-injected mice compared to controls. In the cerebellum, the Fe isotopic composition was significantly lighter in the aged mice and, a shift towards a heavier Fe isotopic composition was observed in the aged LPS-injected mice compared to controls.

Further research is required to establish a potential correlation between the blood plasma Cu and Fe isotopic compositions and the abundance of the corresponding metal-binding proteins, as well as to evaluate the BBB integrity as a potential cause of the observed metal redistribution in brain tissues.

It is however clear that isotopic analysis provides access to information not embedded in element concentrations, thus making both approaches complementary. The complexity of biological systems and the role of isotopic analysis in unraveling the biochemistry in health and disease requires an intense interdisciplinary collaboration.

## Data Availability

The raw data supporting the conclusions of this article will be made available by the authors, without undue reservation.

## Abbreviations

AD, Alzheimer’s disease; ALS, Amyotrophic lateral sclerosis; BBB, Blood-brain barrier; BCSFB, Blood Cerebrospinal fluid barrier; CNS, Central nervous system; CSF, Cerebrospinal fluid; Cp, Ceruloplasmin; PD, Parkinson’s disease; IL, Interleukin; IFN-γ, Interferon-γ; IQR, Interquartile range; LPS, Lipopolysaccharide; MC-ICP-MS, Multicollector-inductively coupled plasma-mass spectrometry; ND, Neurological disorder; PBS, Phosphate buffered saline; ROS, Reactive oxygen species; SAE, Sepsis associated encelopathy; SF-ICP-MS, Sector-field single-collector inductively coupled plasma-mass spectrometry; TNF-α, Tumor necrosis factor.
