# Supplementary material for: High-Precision Isotopic Analysis of Cu and Fe via Multi-Collector Inductively Coupled Plasma-Mass Spectrometry Reveals Lipopolysaccharide-Induced Inflammatory Effects in Blood Plasma and Brain Tissues
Source: Front Chem. 2022 Jun 15;10:896279. doi: 10.3389/fchem.2022.896279 (PMC9241339; doi:10.3389/fchem.2022.896279)
Supplement: Supplementary file 1 [file DataSheet1.PDF]

High-precision isotopic analysis of Cu and Fe via multi-collector inductively coupled plasma-mass spectrometry reveals lipopolysaccharide (LPS)-induced inflammatory effects in blood plasma and brain tissues

Kasper Hobin[1], Marta Costas-Rodríguez[1], Elien Van Wonterghem[2,3], Roosmarijn E. Vandenbroucke[2,3], Frank Vanhaecke[1*]

[1] Atomic & Mass Spectrometry – A&MS research unit, Department of Chemistry, Ghent University, Ghent, Belgium
[2]Barriers in inflammation lab, VIB Center for Inflammation Research, Ghent, Belgium
[3]Department of Biomedical Molecular Biology, Ghent University, Ghent, Belgium

*** Correspondence:**
Corresponding Author
Frank.Vanhaecke@UGent.be

Table S.1: Concentrations and isotope ratios of trace elements Cu and Fe for all plasma samples. 2se is 2 times the standard error.

			Concentrations (μg L^{-1})		Isotope ratios (‰)					
**Sample Type**	**Sample ID**	**Enrollment**	**Cu**	**Fe**	**δ^{65}Cu**	**2se**	**δ^{56}Fe**	**δ^{57}Fe**	**2se**	**2se**
Plasma	Y03	young controls	412	439	-1.12	0.05	-2.12	-3.00	0.04	0.07
Plasma	Y04	young controls	339	461	-1.26	0.09				
Plasma	Y06	young controls	366	578	-1.16	0.12	-2.11	-2.99	0.03	0.05
Plasma	Y01	young controls	684	664	-0.82	0.04	-1.54	-2.11	0.03	0.06
Plasma	Y01-2	young controls	357	1502	-1.39	0.03	-2.24	-3.35	0.04	0.06
Plasma	Y02	young controls	322	564	-1.23	0.03				
Plasma	Y07	young controls	308	869	-1.07	0.03	-2.31	-3.48	0.04	0.06
Plasma	Y08	young controls	272	934	-1.30	0.03	-2.10	-3.15	0.03	0.04
Plasma	Y04	young controls	258	2203	-1.41	0.08	-2.55	-3.31	0.07	0.05

Plasma	Y03	young controls	311	2779	-0.96	0.04	-2.20	-3.21	0.03	0.05
Plasma	Y241	young LPS	455	349	-1.03	0.04	-1.81	-2.62	0.03	0.05
Plasma	Y243	young LPS	561	416	-0.98	0.04	-2.46	-3.34	0.03	0.06
Plasma	Y244	young LPS	464	556	-1.12	0.09	-2.27	-3.29	0.03	0.06
Plasma	Y245	young LPS	515	644	-1.02	0.11	-1.91	-2.65	0.04	0.06
Plasma	Y241	young LPS	576	968	-0.62	0.04				
Plasma	Y24.2-2	young LPS	409	930	-0.74	0.03	-1.66	-2.57	0.03	0.05
Plasma	Y24.7	young LPS	388	841	-0.96	0.03	-2.13	-3.27	0.03	0.07
Plasma	Y24.8	young LPS	559	392	-0.95	0.03	-2.08	-3.24	0.03	0.06
Plasma	Y24.5	young LPS	424	939	-1.06	0.07	-2.44	-3.07	0.02	0.04
Plasma	Y24.6	young LPS	424	1601	-1.02	0.03	-2.00	-2.89	0.04	0.06
Plasma	O01	old controls	363	707	-1.28	0.05	-1.95	-2.79	0.04	0.06
Plasma	O06	old controls	399	455	-1.37	0.04	-2.11	-2.96	0.03	0.05
Plasma	O07	old controls	402	443			-2.01	-2.83	0.03	0.05
Plasma	O01	old controls	402	846	-1.03	0.03	-1.69	-2.34	0.03	0.05
Plasma	O01	old controls	333	455	-1.18	0.03	-2.13	-3.05	0.04	0.06
Plasma	O01-2	old controls	607	651	-0.89	0.03	-2.28	-3.38	0.03	0.05
Plasma	O02.2	old controls	327	534	-1.05	0.03	-2.40	-3.54	0.04	0.06
Plasma	O03	old controls	298	1311	-1.45	0.03	-1.95	-3.05	0.04	0.06
Plasma	O03-2	old controls	366	916	-1.14	0.03	-2.37	-3.61	0.04	0.06
Plasma	O04-2	old controls	363	908	-1.35	0.03	-2.09	-3.12	0.03	0.06
Plasma	O08	old controls	455	344	-0.90	0.07	-2.63	-3.85	0.03	0.06
Plasma	O09	old controls	478	539	-1.09	0.03	-2.28	-3.35	0.03	0.05
Plasma	O010	old controls	335	474	-0.93	0.03	-1.78	-2.68	0.03	0.05
Plasma	O 04	old controls	322	1060	-1.57	0.02	-2.50	-3.26	0.16	0.20
Plasma	O0.3	old controls	427	1326	-1.25	0.03	-2.46	-3.62	0.04	0.06
Plasma	O241	old LPS	533	437	-0.50	0.05	-1.66	-2.38	0.03	0.06
Plasma	O244	old LPS	480	501	-0.54	0.05	-1.68	-2.43	0.04	0.08
Plasma	O245	old LPS	421	1233	-0.73	0.05	-1.81	-2.46	0.03	0.06
Plasma	O047	old LPS	951	564	-0.80	0.04	-1.91	-2.59	0.06	0.08
Plasma	O242	old LPS	595	1098	-0.80	0.06	-2.07	-2.76	0.03	0.06

Plasma	O24.2-2	old LPS	497	600	-0.83	0.04	-2.09	-3.22	0.04	0.06
Plasma	O24.4-2	old LPS	531	1611	-0.58	0.03	-1.83	-2.76	0.04	0.04
Plasma	O24.9	old LPS	408	954	-0.75	0.03	-1.86	-2.72	0.04	0.05
Plasma	O 24.5	old LPS	360	2772	-0.79	0.03	-2.53	-3.05	0.03	0.06
Plasma	O24.4	old LPS	317	4851	-0.65	0.06	-2.00	-2.81	0.04	0.07

Table S.2: Concentrations and isotope ratios of trace elements Cu and Fe for all brain tissue samples. 2se is 2 times the standard error.

			Concentrations (µg g-1)		Isotope ratios (‰)					
Sample Type	Sample ID	Enrollment	Cu	Fe	δ^{65}Cu	2se	δ^{56}Fe	δ^{57}Fe	2se	2se
Tissue	Y01 hippocampus	young controls	4	8	0.54	0.07				
Tissue	Y01 cortex	young controls	3	7	0.17	0.03	-1.05	-1.52	0.03	0.06
Tissue	Y01 brain stem	young controls	3	5	0.78	0.03	-1.79	-1.85	0.03	0.06
Tissue	Y01 cerebellum	young controls	5	6	0.47	0.03				
Tissue	Y01 hippocampus	young controls	3	3	0.36	0.05				
Tissue	Y01 cortex	young controls	3	4	0.30	0.04	-2.66	-3.76	0.02	0.04
Tissue	Y01 brain stem	young controls	4	3	0.42	0.02	-2.07	-3.03	0.03	0.04
Tissue	Y01 cerebellum	young controls	5	23	0.40	0.03	-2.22	-3.21	0.03	0.05
Tissue	Y04 hippocampus	young controls	2	6	0.03	0.04	-2.41	-2.88	0.03	0.05
Tissue	Y04 cortex	young controls	2	9	0.03	0.02	-2.20	-3.04	0.11	0.13
Tissue	Y04 brain stem	young controls	2	6	0.25	0.07	-1.55	-2.00	0.16	0.09
Tissue	Y04 cerebellum	young controls	4	10	0.06	0.05	-2.09	-2.93	0.10	0.17
Tissue	Y 02 hippocampus	young controls	8	6	0.10	0.03				
Tissue	Y02 cortex	young controls	4	3	0.07	0.05				
Tissue	Y02 brain stem	young controls	4	3	0.39	0.02				
Tissue	Y02 cerebellum	young controls	5	3	-0.02	0.01				
Tissue	Y03 hippocampus	young controls	4	16	0.34	0.04	-2.14	-3.08	0.04	0.07
Tissue	Y03 cortex	young controls	3	14	0.20	0.03	-2.17	-3.15	0.03	0.06
Tissue	Y03 brain stem	young controls	3	15	0.59	0.03	-2.10	-3.10	0.04	0.07
Tissue	Y03 cerebellum	young controls	5	14	0.62	0.04	-2.10	-3.11	0.03	0.06
Tissue	Y241 hippocampus	young LPS	3	4	0.54	0.03				
Tissue	Y241 cortex	young LPS	3	4	0.45	0.04	-2.06	-2.98	0.04	0.07

Tissue	Y241 brain stem	young LPS	3	4	0.87	0.03	-1.82	-2.49	0.03	0.06
Tissue	Y241 cerebellum	young LPS	5	4	0.42	0.04	-2.05	-2.91	0.03	0.06
Tissue	Y24.1 hippocampus	young LPS	4	4	0.29	0.05	-2.63	-3.45	0.02	0.05
Tissue	Y24.1 cortex	young LPS	4	3	0.01	0.04	-2.32	-3.25	0.03	0.04
Tissue	Y24.1 brain stem	young LPS	3	4	0.02	0.03	-2.42	-3.51	0.02	0.05
Tissue	Y24.1 cerebellum	young LPS	5	4	-0.01	0.03	-2.19	-3.19	0.07	0.15
Tissue	Y24.5 hippocampus	young LPS	3	9	0.27	0.12	-2.19	-2.66	0.18	0.24
Tissue	Y24.5 cortex	young LPS	3	9	-0.01	0.03	-2.18	-2.90	0.08	0.12
Tissue	Y24.5 brain stem	young LPS	3	9	0.38	0.02	-2.22	-2.99	0.10	0.12
Tissue	Y24.5 cerebellum	young LPS	4	14	0.24	0.03	-1.89	-2.61	0.04	0.09
Tissue	Y24.6 hippocampus	young LPS	3	13	0.32	0.03	-2.13	-3.13	0.04	0.06
Tissue	Y24.6 cortex	young LPS	2	13	-0.07	0.04	-2.04	-3.00	0.04	0.06
Tissue	Y24.6 brain stem	young LPS	3	11	0.47	0.03	-2.11	-3.09	0.03	0.05
Tissue	Y24.6 cerebellum	young LPS	5	14	0.53	0.03	-2.14	-3.14	0.03	0.06
Tissue	Y24.2 hippocampus	young LPS	4	3	0.46	0.07	-1.88	-1.91	0.04	0.08
Tissue	Y24.2 cortex	young LPS	5	5	0.17	0.06	-1.68	-2.28	0.03	0.07
Tissue	Y24.2 brain stem	young LPS	4	4	0.37	0.04	-2.14	-3.16	0.05	0.06
Tissue	Y24.2 cerebellum	young LPS	5	4	0.25	0.04	-2.22	-3.29	0.03	0.05
Tissue	O01 hippocampus	old controls	3	9	0.38	0.04	-2.03	-2.85	0.03	0.04
Tissue	O01 cortex	old controls	3	5	0.22	0.04	-2.31	-3.20	0.03	0.05
Tissue	O01 brain stem	old controls	3	4	0.62	0.04				
Tissue	O01 cerebellum	old controls	7	7	0.46	0.04	-2.54	-3.63	0.07	0.20
Tissue	O03 hippocampus	old controls	4	14						
Tissue	O03 cortex	old controls	6	4	0.06	0.03	-2.55	-3.70	0.03	0.02
Tissue	O03 brain stem	old controls	4	4	0.51	0.03	-2.19	-3.20	0.02	0.04
Tissue	O03 cerebellum	old controls	3	4	0.10	0.04	-3.01	-4.33	0.02	0.04
Tissue	O04 hippocampus	old controls	3	4						
Tissue	O04 cortex	old controls	3	4	0.37	0.05				
Tissue	O04 brain stem	old controls	3	4	0.09	0.03	-2.19	-3.27	0.02	0.04
Tissue	O04 cerebellum	old controls	6	5	0.17	0.03	-2.22	-3.27	0.02	0.05

Tissue	O 04 hippocampus	old controls	3	8	0.27	0.04	-2.54	-2.93	0.03	0.05
Tissue	O 04 cortex	old controls	2	9	0.11	0.06	-2.29	-3.07	0.07	0.07
Tissue	O 04 brain stem	old controls	3	4	0.26	0.04	-1.78	-2.22	0.09	0.01
Tissue	O 04 cerebellum	old controls	6	11	0.25	0.01	-2.50	-3.52	0.07	0.06
Tissue	O 03 hippocampus	old controls	2	14	0.26	0.04				
Tissue	O 03 cortex	old controls	2	12	0.06	0.03	-2.36	-3.44	0.04	0.06
Tissue	O 03 brain stem	old controls	3	11	0.33	0.03	-2.32	-3.46	0.03	0.06
Tissue	O 03 cerebellum	old controls	6	16	0.58	0.03	-2.49	-3.68	0.03	0.06
Tissue	O242 hippocampus	old LPS	5	6	0.38	0.04	-1.93	-2.57	0.02	0.05
Tissue	O242 cortex	old LPS	3	5	0.21	0.04	-1.96	-2.74	0.03	0.05
Tissue	O242 brain stem	old LPS	4	4	0.33	0.04	-1.88	-2.60	0.03	0.05
Tissue	O242 cerebellum	old LPS	7	6	0.08	0.03	-2.31	-3.37	0.05	0.05
Tissue	O24.2 hippocampus	old LPS	3	3	0.32	0.03	-2.86	-4.20	0.02	0.04
Tissue	O24.2. cortex	old LPS	4	3	-0.03	0.05	-2.73	-3.76	0.03	0.04
Tissue	O24.2 brain stem	old LPS	3	3	0.14	0.03	-2.28	-3.39	0.02	0.04
Tissue	O24.2 cerebellum	old LPS	6	4	0.24	0.03	-2.43	-3.53	0.01	0.03
Tissue	O24.4 hippocampus	old LPS	4	3	0.30	0.04	-2.94	-4.10	0.02	0.04
Tissue	O24.4 cortex	old LPS	3	4	0.03	0.03	-2.44	-3.62	0.02	0.04
Tissue	O24.4 brain stem	old LPS	4	4	0.33	0.04	-2.48	-3.55	0.03	0.05
Tissue	O24.4 cerebellum	old LPS	7	5	0.35	0.03	-2.35	-3.46	0.02	0.04
Tissue	O 24.5 hippocampus	old LPS	11	20	0.61	0.06	-2.79	-3.90	0.19	0.33
Tissue	O 24.5 cortex	old LPS	3	16	0.35	0.02	-1.56	-2.09	0.08	0.10
Tissue	O 24.5 brain stem	old LPS	2	4	0.67	0.07	-2.25	-2.86	0.04	0.06
Tissue	O 24.5 cerebellum	old LPS	1	6	0.53	0.03	-2.60	-3.29	0.11	0.12
Tissue	O 24.4 hippocampus	old LPS	4	18	-0.18	0.04	-2.40	-3.47	0.03	0.05
Tissue	O 24.4 cortex	old LPS	3	17	0.14	0.04	-2.20	-3.22	0.03	0.05
Tissue	O 24.4 brain stem	old LPS	3	13	0.02	0.02	-2.43	-3.56	0.03	0.05
Tissue	O 24.4 cerebellum	old LPS	5	12	-0.17	0.04	-2.35	-3.43	0.04	0.06
